# “That’s all Fake”: Health professionals stigma and physical healthcare of people living with Serious Mental Illness

**DOI:** 10.1371/journal.pone.0226401

**Published:** 2019-12-18

**Authors:** Eliut Rivera-Segarra, Nelson Varas-Díaz, Axel Santos-Figueroa

**Affiliations:** 1 School of Behavioral and Brain Sciences, Ponce Health Sciences University, Ponce, Puerto Rico; 2 Department of Global and Sociocultural Studies, Florida International University, Miami, United States of America; 3 Department of Psychology, University of Puerto Rico, Mayagüez Campus, Mayagüez, Puerto Rico; Emory University School of Public Health, UNITED STATES

## Abstract

**Background:**

People living with a Serious Mental Illness (SMI) die earlier than the general population due to preventable medical conditions. Latinos living with SMI are a particularly vulnerable population with higher prevalence of chronic medical conditions. Stigma has been identified as a factor that fosters health inequities for Latinos/as with SMI, particularly Puerto Ricans. Although personal and social consequences of stigmatization have been well documented, research regarding the role of cultural factors on healthcare interactions is scarce. Furthermore, little research has focused on addressing stigma from the perspective of healthcare professionals.

**Methods:**

We investigated this process through a qualitative design using semi-structured individual interviews with 11 healthcare professionals (8 physicians and 3 nurses) in Puerto Rico. We conducted a thematic analysis to analyze the data.

**Results:**

Following a thematic analysis, we found three main themes and nine sub-themes related to the stigmatization process. Some participants reported perceptions of dangerousness and uneasiness, social distance and inadequate care. Participants also emphasized a lack of medical training regarding SMI within the Puerto Rican context.

**Conclusions:**

These findings support the need to develop culturally appropriate public health interventions targeting healthcare professionals in order to address stigma and eliminate health disparities among Latinos/as with SMI.

## Background

Serious Mental Illness (SMI) is defined as mental behavioral or emotional disorder resulting in functional impairment that substantially interferes with or limits one or more major life activities [1]. Schizophrenia, Bipolar Disorder and Major Depressive Disorders are the most commonly associated diagnoses associated with SMI. Research has documented that people diagnosed with a Serious Mental Illness (PWSMI) die on average, 25 years younger than the general population [2–4]. Research has documented how preventable medical conditions such as cardiovascular disease and diabetes account for the high mortality among this population [5–7]. Latinos/as diagnosed with Serious Mental Illness (SMI) are a particularly vulnerable population. They have been identified with a higher prevalence of chronic medical conditions such as cardiovascular disease and diabetes than non-Latinos/as with SMI’s [8–10]. This scenario worsens for one particular Latino/a sub-group, Puerto Ricans. Puerto Ricans are U.S. born citizens who have an estimate prevalence of up to 7.3% of SMI when living in the Caribbean archipelago [11,12], and the highest prevalence of mental illness among the Latino/a communities in the mainland U.S. [13,14]. Simultaneously, they hold one of the highest rates of cardiovascular disease and diabetes among all Latino/a communities [15–17]. Despite this alarming public health issue, efforts to examine the psychosocial factors that foster these health inequities among this made vulnerable population have been largely absent from the scientific literature.

### Stigma as a key social determinant of health

Stigma has been identified as an overlooked social determinant of health that plays a vital role in the distribution of life chances and health status for PWSMI through the production of inequities and stress [18–20]. Stigmatization is a complex process of social control in which labeling, stereotyping and negative attitudes towards a person, based on a condition or behavior often lead to status loss, rejection or discrimination [21–23]. Stigma is also a multilevel process that occurs at the individual, interpersonal, and/or structural level [24]. At the innermost level are the individuals and their intrapersonal dynamics such as emotions, cognitions and experiences. At the interpersonal level are the interactions between two or more individuals, which can happen in any given social context from family to healthcare clinical services. Finally, the structural level refers to the larger social structure such as institutions or organizations that can affect material resources, policies, practices, etc. This multilevel process suggests that relationships between individuals, society and larger societal structures are intertwined and influence each other within and between levels [24]. Furthermore, although stigmas’ multilevel process appears to be universal with similar features across cultures (i.e. negative perceptions) [25–27], their manifestations and specific experiences are local and influenced by the cultural context [28,29].

### Stigma, mental illness and culture

Literature has extensively documented stigmatization towards PWSMI, particularly among PWSMI and the general population. For example, research has documented increased levels of social distance desire, perceptions of dangerousness, and attitudes of blame and fear towards PWSMI among the general population [30–32]. Furthermore, these negative attitudes among the general population are often internalized by PWSMI, who then depict themselves in a similarly negative fashion [33–35], leading them to a sense of social defeat or “why try effect” that has direct negative consequences on their life goals and general health [36,37]. However, most of these findings focus on North America and Europe neglecting to address how the stigmatization of PWSMI is manifested in different cultural contexts, such as Latin America and the Caribbean [38,39].

Recent literature suggests that several features and values within the Latino/a culture might play an important role in the stigmatization process of PWSMI in this context [39–42]. For example, *familismo* is a common core value within the Latino/a culture that places a strong emphasis on the identification, attachment, and obligation to nuclear and extended family members [43,44]. Although traditionally seen as a social support system, recent literature suggests it can also impact in negative ways. Specifically, *familismo* can function in Latino/a culture as a mechanism for PWSMI to refrain from discussing their illness outside their family environment in order to avoid the shame and social exclusion attached to mental illness from being connected to their family [41,44]. *Machismo* is another common value within the Latino/a culture associated with the process of stigmatization [40,42]. *Machismo* refers to a complex cultural value characterized by normative ideas about masculinity (i.e. chivalry, dominance or honor) [45,46] and it has been documented how it leads PWSMI to perceive healthcare services as something for weak men but not for “real men” [40]. Finally, *religion* can also be seen as another cultural factor linked to the stigmatization process of PWSMI within the Latino/a cultural context. This is particularly relevant for Judeo-Christian related religions due in part to the conceptualization of several mental illness symptoms as sins (i.e. suicide attempts or drug abuse) [47]. In addition, it can also impact PWSMI’s treatment seeking behaviors through the belief that healthcare is not needed in light of their religion [40]. Thus, recent literature evidences the need to address the role of cultural values that “matter most” within the Latino/a communities while also suggesting the importance of focusing on other fundamental actors in social interactions within the healthcare system such as Latino/a healthcare professionals [39, 42, 48].

### Mental illness stigma and healthcare professionals

Healthcare professionals are an integral part of the formal healthcare service system who have been identified as an underrepresented group within the SMI stigma literature [28, 49–52]. Evidence suggests that healthcare professionals share similar attitudes to those of the general population perceiving PWSMI as incompetent, violent, and dangerous [53–55]. For example, research has shown that health professionals have more negative stereotypes and social distance desire for people with schizophrenia than people with depression [56–58]. Similarly, providers seem to perceive people with bipolar disorder as dangerous and unpredictable [59–60]. Recent evidence has also suggested that professionals often perceive people with borderline personality disorder as manipulative and less deserving of care [61]. Other recent findings have also found similarly negative attitudes and emotions towards people with suicide ideations or attempts [62,63]. Finally, research has also documented that healthcare professionals have lower regards towards patients with comorbid mental illness and substance abuse [64–67]. Thus, recent research has suggested the need to target healthcare professionals in order to address stigmatization in healthcare facilities and its implications for people with SMI physical healthcare [68–69]. This is a troublesome issue, particularly within the Latino/a cultural context. If PWSMI’s main avenue for entering the pipeline of care becomes a breeding ground for stigmatizing attitudes and behaviors, the quality of the healthcare services they receive can become negatively affected or in some instances completely ignored. Alarmingly, recent research in the Puerto Rican context has also found stigmatizing attitudes towards PWSMI, including drug abuse and suicide among healthcare professionals in training, particularly among physicians in training [62,67,70]. However, in order to develop culturally sensitive stigma reduction interventions, there is still a need to understand how stigma attitudes towards PWSMI are manifested from the perspective of healthcare professionals who are currently in the field within the Puerto Rican context. Thus, the objective of this research was to document stigma attitudes towards SMI among practicing healthcare professionals in Puerto Rico.

## Method

### Design

Given the exploratory nature of our study and recent recommendations to better understand social inequities in health [71], we implemented a qualitative design. Recruitment was completed using a purposive sampling. We conducted 11 single in-depth semi-structured interviews among practicing healthcare professionals in Puerto Rico in order to gain a deeper understanding of the stigmatization process in their healthcare interactions with PWSMI [72].

### Participants

Participants in this study consisted of 11 practicing healthcare professionals (eight physicians and three nurses) in Puerto Rico who agreed to participate in individual interviews. Providers in the study were not in charge of patient’s routine mental healthcare, but rather were focused on addressing physical healthcare. The selection of physicians and nurses was informed by recent literature suggesting high levels of stigma among these two specific professions [70,73]. All participants were practicing their profession in Puerto Rico and providing services in inpatient and outpatient medical institutions located in the metropolitan areas of the Island. Participants were mostly men (n = 6), married (n = 6) and full time employed (n = 10). In addition, all identified themselves as religious. For a detailed summary of the participant’s socio-demographic information refer to Table 1.

**Table 1:** Socio-demographic information.

Variable	Frequency
Age	
21–30	2
31–40	3
41–50	1
51–60	4
> 61	1
Gender	
Male	6
Female	5
Civil Status	
Married	6
Single	5
Religious Affiliation	
Catholic	9
Pentecostal	1
Baptist	1
Importance of Religion	
Important	9
Very Important	2
Employment	
Full-time	10
Part-time	1
Annual Income	
Less than 30,000	1
30,001–40,000	3
50,001–60,000	1
More than 70,000	6
Health Related Field	
General Medicine	5
Internal Medicine	3
Nursing	3
Years in Practice	
1–10	4
11–20	3
21–30	2
>30	2

Note: N = 11

### Procedure

The research protocol for this study was evaluated and approved by the Ponce Health Sciences University Institutional Review Board (IRB). Following approval, we began the recruitment process by distributing promotion sheets to key personnel such as medicine and nursing program directors, faculty at medical schools, as well as medical faculty in several general hospitals and community centers in Puerto Rico. Once potential participants contacted the first author, a schedule for the interviews was agreed at the participant’s preferred time and place. The first author did not had any relationship with participants and participants were informed about the reasons for conducting the study during the consent. Although no participant refused to participate in the study or dropped out, all participants requested for the interviews to be conducted in a private office within their respective practicing healthcare scenarios. No one else was present during the interviews. All interviews were conducted in Spanish. Participants in this study did not receive any compensation. No field notes were made during the interviews or after them.

The individual interviews were audiotaped and conducted face-to-face during the period of November 2014 and February 2015 by the first author a current PhD, who was an advance graduate student in Clinical Psychology at the time, identified himself as a man and had previous training and experience conducting in-depth and semi-structured interviews [74]. Each interview lasted approximately one hour. After completing one interview, the first author proceeded to transcribe that interview and remove all potential identifiers [75]. In order to maintain rigor and validity during the interview process, the first author met with the second and third authors for a debriefing after each interview to address issues such as: under emphasized areas, specific techniques to manage the interviewer’s emotions related to the interview setting and management of power dynamics in the interview encounter, among others [76, 77]. After the debriefing, the first author proceeded to conduct the next scheduled interview. This process continued until we achieved data saturation [78] and then proceeded to conduct data analysis. Transcripts were not returned to participants for comments or corrections.

### Data analysis

Our analysis was informed by Braun and Clarke's [79] step-by-step guide for conducting thematic analysis. We were also guided by Cook and colleagues (2014) conceptualization of stigma as a multilevel process and Yang and colleagues (2007) emphasis on the contextual and cultural specificity of the stigmatization process [24,29]. Both frameworks informed our data analysis process from the development of themes to the writing of the report.

We began our hybrid inductive and deductive [80] manual process of analysis familiarizing ourselves with the data in order to ensure its fidelity by repeated individual reading of the transcripts without any computer software’s. Simultaneously, all authors individually generated initial themes from the data. After this process we met weekly for a period of two months working systematically through the transcripts and discussing our individual analyses to ensure their trustworthiness. After identifying our initial themes and sub-themes we developed a thematic map in order to better think about their relationship with the text extracts and the theory. We did not search for patterns (frequency), as they do not necessarily reflect the most important themes [79]. After reaching consensus on the quotations to be included in our final report [81], the first author translated from Spanish to English all quotations. The second and third authors validated the translations in order to begin the process of writing the report. Due to our limited sample size and the limited number of private institutions in the Island we will present quotations solely focusing on the participants disciplines in order to secure participants identities.

## Results

We organized our findings into three main themes related to the multilevel process at which social stigmatization occurs and eight sub-themes focused on the stigma drivers or manifestations (Table 2)

**Table 2:** Themes and sub-themes descriptions.

Themes/Sub-themes	Descriptions
1. Individual Level	Includes quotations regarding healthcare professional’s cognitive representations of PWSMI.
1.1 Negative Perceptions	Includes examples of thoughts and negative stereotypes about PWSMI.
1.2 Blaming the Patient	Includes examples of thoughts regarding patient’s responsibility about their physical health.
1.3 Inability to Recover	Includes examples of thoughts regarding PWSMI recovery and societal integration.
2. Interpersonal Level	Includes quotations related to clinical interactions experiences with PWSMI in medical healthcare scenarios.
2.1 Diagnostic Overshadowing	Includes examples in which the patient’s symptoms are neglected or misattributed to their SMI.
2.2 Lack of Skills	Includes examples about how lack of skills can drive negative patient-provider interactions.
2.3 SMI Diagnosis Disclosure	Includes examples of patient’s lack of disclosure or professional’s inquiry avoidance in clinical interactions.
3. Structural Level	Includes quotations regarding participant’s perceptions and interactions with society’s health-related institutions.
3.1 Lack of Training	Includes examples of their perceptions regarding how medical training experiences foster or hampers their ability to address PWSMI healthcare issues.
3.2 Healthcare Systems	Includes examples related to their perceptions and experiences with how the healthcare system fosters or hampers healthcare services for PWSMI in Puerto Rico.

### Individual level

The discussion about the individual stigma level focused on healthcare professional’s cognitive representations of PWMSI. We found three related sub-themes that drove these cognitive representations at the individual level: (1) negative perceptions, (2) blaming the patient, and (3) inability to recover. We present each sub-theme in the next paragraphs.

#### Negative perceptions

One of the most consistent findings during the interview discussions was the negative perceptions surrounding PWSMI among the participants. When defining or describing PWSMI, participants mostly depicted them as social outliers.

A person we call antisocial, or that society has categorized as an individual that does not do what someone must usually do within a society…For example, following certain rules… [a person that] is not governed by a certain religion, not governed by the rules of society. (Generalist—1)

Along the same line, other participant echoed these descriptions and further explained how this status as social outliers led to his view that PWSMI are generally problematic in their daily social lives: “A patient with mental illness tends to have many problems in society, they tend to have many problems with family members, their neighbors, and problems all the time wherever they go” (Generalist—9).

Interestingly, another participant linked the negative stereotype of PWMSI as socially problematic with the perception of them being from the center or the rural parts of the Island. This perception is somewhat widespread among some Puerto Ricans, particularly those living in metropolitan areas, who can use the term “those from the Island” as a pejorative phrase to refer to people living outside the metropolitan areas perceived to be social outliers such as those from low socio-economic class or low educational level. One representative example in which PWSMI were perceived as belonging into this category is presented in the following quotation:

Many people with mental illness live specifically in the centermost part of the island … and some of their problems both for schizophrenics and bipolars, is having problems within the family. Some families live there together with one another, and often their families do not love each other. The same family will try to kill each other and that's part of psychiatric conditions… they are people who do not have much, they are not people who have a lot of education, they are not very educated, they don’t work. They instead have many bars to drink, dedicate themselves to drink, and because education is part of controlling mental conditions, if they don’t have it, obviously it is easy to get out of control … Educated people are more protected from psychiatric traits because they are aware of what are some psychiatric conditions. (Generalist—6)

Some participants emphasized that negative perceptions about PWMSI are not only widespread among the general population, but also within health professionals. One participant explained the urgency of dealing with these negative perceptions among the medical personnel in order to address effectively PWSMI medical problems:

The stereotype they have as patients, for example, they are in X psychiatric hospital, and any patient that has been there, is already crazy. That must be stopped … they are patients like any other with another type of diagnosis. The only thing is that their diagnosis has nothing to do with the pancreas; it has to do with their head. (Internist—8)

#### Blaming the patient

In this sub-theme the quotations exemplified the perception that PWSMI are solely responsible for their healthcare. As one participant mentioned, the main issue seems to be that the patients do not follow up or seek medical attention because it is not one of their priorities and this issue is the one that leads them to have poor health:

They do not monitor themselves… It’s not their priority because they do not routinely monitor themselves to see how their other conditions evolve … They don’t do it for themselves, because you cannot go to the patient’s house. The patient is the one that has to come to us basically and although you advise them they are going to make their own decisions … It’s the person. (Internist—7)

Another participant echoed this and further detailed that their lack of knowledge could also be behind their frequent physical health issues.

If all of them don’t know how to handle things behaviorally and mentally, then obviously problems begin with their blood pressure, food intake, substance problems, cholesterol, and they can also develop cardiovascular problems. (Generalist– 2)

#### Inability to recover

Another finding during the discussions focused on participant’s perceptions regarding the course of mental health treatment for PWSMI. Their thoughts around this topic emphasize a pessimistic view regarding recovery for this population. As one participant clearly stated: “They are not curable. It’s for their whole lives. They can get better with therapy or medication… but they will not be cured.” (Generalist—1).

This perception echoed by another participant who further explained the implications of PWSMI being unable to recover in terms of management and potential integration into society. As this participant stated:

I believe they are not suited to handle certain situations. They are not mentally prepared people … I consider that there are patients that should not be in their homes… Therefore, they should be in institutions that can deal… that have the preparation to deal with those kinds of patients… Maybe in a home, I don’t know, where they could be like admitted long-term. (Nurse—4)

### Interpersonal level

During our discussion, participants focused on their clinical interactions experiences with PWSMI in their routine medical healthcare scenarios. We found four related sub-themes that seemed to guide interpersonal level stigma in healthcare scenarios: (1) diagnostic overshadowing, (2) fear and dangerousness, (3) treatment complexity, and (4) SMI diagnosis disclosure difficulties. We present each sub dimension in the next paragraphs.

#### Diagnostic overshadowing

A main finding in this sub-theme was the emphasis on how sometimes healthcare providers misattributed PWSMI physical symptoms due to their mental health diagnosis. One participant went even further and linked this issue with PWSMI mortality.

If a patient goes and says I am seeing things, hearing things, it immediately means they are making it up or he is crazy, they do not take him seriously…‘That’s all fake’ (“Eso es de embuste”). When they come to their doctors, if they have one, as soon as they say they are diagnosed with a serious mental illness everything they say is like, not true at all. ‘Look he complains about chest pain.’ ‘Ah! That’s all in their mind’. They won’t give them the appropriate diagnosis, the appropriate examinations, because all that the patient says from that moment onward is fictitious … There are patients that might be looking for attention, but there are others that have some symptom and are not treated adequately. Little by little it does harm to their systems and possible might be the cause of their deaths. (Internist—8)

Interestingly, another participant also echoed this link between neglect of physical symptoms and potential disease among PWSMI. “They focus only on the mental issues and they leave the physical behind, and then they deteriorate.” (Nurse—11).

In addition, another participant emphasized how patient’s complaints could be neglected via referral’s psychiatrists. In this example quote, the participant mentioned that, in the end, the treatment of PWSMI was not within the medical setting but within the psychiatric setting:

Honestly, we do nothing… we put them on medication to try to control them so that they let us do the laboratories and then send them to another place. A psychiatric hospital where they will get the final treatment for that patient. (Generalist—6)

#### Lack of Skills

Another important finding stems from participant’s discussion regarding their skills and behaviors in their routine clinical interactions, which were also linked to their thoughts and emotions about PWSMI. This discussion evidenced how lack of skills might impact their healthcare interactions with PWMSI. For example, one participant mentioned a desire for social distance in the interactions as follow:

Sometimes even within the medical faculty they do not want to interact much with those conditions. I think that maybe they are afraid, I don’t know, about the topic. Well, maybe they are not well informed about it. (Internist—7).

Another participant also echoed these thoughts and brought examples from clinical practice in which emotions can lead to negative patient-provider clinical interactions, in this case a rushing a patient into the physician’s office.

Still today, I mention the word schizophrenia anywhere and nurses get… they are so afraid of the patients that they will take them into my office in a second, in a second. That is, the patient enters the clinic’s doors and they pass them quickly directly into my office. It has happened to me and continues to happen … Also, the other patients become super crazy… “look at him ‘ten cuidao '‘ (be careful), pass him in”. They are also afraid… (Generalist—9)

Another participant also emphasized the avoidance by healthcare staff and how dangerousness can play a key role in terms of their clinical interactions and patient’s treatment (or lack of it):

Sometimes we put them in an isolated room and restrict them, they are guarded, and they have a person that watches over them, a guard… We should not put them a label just because they are mental, but we need to remove them … Its not to isolate them, its for security issues. (Nurse—11)

#### SMI diagnosis disclosure difficulties

Another finding evidenced one of the difficulties in terms of communication in their healthcare interactions with PWSMI was patient’s lack of disclosure of their SMI diagnosis. Healthcare professionals discussed consistently these experiences in their clinical practices: “When one asks them about their medical conditions, they suffer from X and Y condition, but they never mention the mental part until one asks them.” (Internist—2). Another participant echoed this experience and expressed how to foster patient’s mental illness disclosure:

In my case, I give them an open-ended question about their conditions… Many times patients do not believe a condition is a condition, and then I proceed in an indirect manner to ask them ‘What medications do you use? For what do you use that medication?’ I do not tell them that that medication is for schizophrenia; I ask them for what do they use that medication and they have to tell me. (Generalist—6)

Despite the importance of disclosing one’s mental illness status in a healthcare scenario, some healthcare professionals openly mentioned that they did not asked them about their mental health history “I do not ask them so I don’t make them feel-…Because they are fearful of saying they are bipolar, that they are a person with mental health problems.” (Nurse—11). Another participant discussed that a negative feeling associated with disclosing SMI diagnosis could be a potential reason behind a patient’s disclosure difficulties with healthcare professionals. In addition, this participant provides a quote that exemplifies not only difficulties with patient’s lack of disclosure, but also difficulties from healthcare professional’s side to ask about them.

Patients that have tried to suicide, maybe when they arrive they do not want to tell me about it at the beginning because they might fear about being labeled as a mentally unstable person or X or Y. It hasn’t happened, well, that I know of. Maybe they haven’t told me, I just hope it hasn’t happened. (Internist -7)

### Structural level

Our final theme focuses on the participants’ perception and experiences regarding how the macro-level aspects of the stigma process, specifically with health-related care institutions. We identified two sub-themes: (1) lack of training and (2) healthcare systems.

#### Lack of training system

Participants discussed the lack of appropriate formal training for working with PWSMI and how to address their healthcare issues. Some participants even expressed they had not received any training at all from their medical education institutions:

In terms of training, medical school doesn’t really help at all. What helps is the experience… In training the only thing they teach you is that you cannot hit a schizophrenic patient, one can only ask for help. Basically you cannot do anything, that’s my training. (Generalist—6)

Other participants discussed they had some, but limited, training. Some of them explained how this might account for the emotional difficulties in their interactions with PWSMI: “Maybe we are not exposed to psychiatric patients as much. I believe that lack of exposure generates a shock when you encounter these patients.” (Internist—7).

Along the same lines, other participants exposed the challenges this creates for the upper medical training years, further specialized training, continuing education and beyond. They emphasize the poor preparation that continued education provides in order to address PWMSI issues: “Continued education courses are very general. They may say something about it, but very little. It is mostly through experience and reading. (Generalist—5)

In all my continued education courses, I always take psychological aspects of illness… but module after module that I receive; it’s just a ‘copy and paste’. I have not seen any change at all. They are not teaching anything new. (Nurse—4)

#### Healthcare system

Participants also described their experiences with the healthcare system in Puerto Rico, specifically when working with PWSMI’s. Most of these experiences were described as negative. One participant explained that the difficulties they encounter when navigating services focused on their SMI, as opposed to physical illnesses are markedly different, evidencing to some extent, the exclusion of this population:

Most of the time, psychological or psychiatric services in Puerto Rico are rather difficult … If we send a patient to the office, they really do not need hospitalization. If you facilitate psychiatric services, rather than refer them to a psychiatric hospital, control them in the office…The system, the appointment system, the medical part, the psychiatric medical part cannot be similar to an emergency system. If the patients feel ill, they should have the availability to reach their psychiatrists if available, in an office … you know I have worked in a hospital and have called psychiatry services at another place to send them there, but often they do not want them. And we all have the same system, they are full! ‘We cannot take them in now. You have to wait another 24 hours and check again’. They make it a little difficult for you.” (Generalist—6)

Although this might appear as a distal aspect to address, another participant stated the direct implications it had for the physical health of PWSMI as well as healthcare professional’s respective practices:

They don’t pay much attention to psychiatric illnesses… they do not check the lithium in three years and then when they come back, the patient has diabetes, renal problems, thyroid problems, basically they don’t do follow-ups… It will have to be Medicare that tells them ‘we are going to penalize you by not paying you for the visit’ or ‘we are going to help you, give you an incentive if you do the follow ups’. Everything works with money. (Internist—8)

Another participant addressed other issues that show the structural inequities encountered by PWSMI in healthcare scenarios and community centers.

There are many limitations (within a general hospital). We only have two social workers and sometimes you need to call APS (State public insurance) and wait for approval. If they have private insurance they need to go to another place, and that takes so much time… everything gets delayed and when the patients feel a bit better… they break out. (Nurse—11)

Despite these barriers, some healthcare professionals mentioned how they dealt with these structural complexities at their individual practices. Some of them mentioned their difficulties and in some cases despair at how institutional policies are reflected in their daily medical interactions. One example is the following:

One of the main problems I see is that there’s no communication between the primary physician and the people that treat mental health patients. In my case I have had many problems. I do home visits and give the patients my cell phone number. If there’s a psychiatrist, psychologist or specialist I tell them ‘call me, there’s my cell phone number if they have something to tell me’. That is very difficult, I have never heard from them. Only one specialist wrote his phone number in the referral form and wrote ‘If you have doubts please call me, I will explain further’. I believe we are not practicing medicine, as it should be…I try to give my 100 percent, but the more I try I definitively cannot do it alone. We need all the help we can get from everywhere…I feel that my hands are tied. (Generalist—9)

## Discussion

Our results suggest the importance of addressing stigmatization as a multilevel process with universal features that is experienced and manifested within specific local and cultural contexts. Healthcare professionals are an important group to target in these endeavors in order to address the impact of stigmatization and its implications for the healthcare of PWSMI. They lie at an intersection in which they can simultaneously enforce structural policies through face-to-face interactions with PSWMI. Focusing on practicing healthcare professionals allows for a deeper understanding of how this process is linked, produced, and reproduced in specific cultural contexts and interpersonal interactions such as healthcare encounters [82]. This has recently been acknowledged by research documenting how stigma attitudes might negatively impact provider’s healthcare decisions when treating PWSMI [83]. In this study, we aimed to contribute to this emerging literature by focusing on how stigma is manifested specifically in healthcare professional’s interactions with PWSMI. Following recent literature recommendations to address health disparities [71], we gathered a more in-depth and contextualized understanding through the lens of practicing healthcare professionals in the Puerto Rican context. This allowed us to understand how perceptions, attitudes, behaviors, and local institutional policies contribute to the stigmatization process in clinical care interactions in Puerto Rico. Although many interpretations might emerge from our findings, there are some salient areas that we would like to discuss.

One of our main results suggests that stigmatization has the potential to play an important role on these health disparities among PWSMI in Puerto Rico. Consistent with the epidemiological data found in other cultural contexts [84,85], most of the participants offered examples of many cases encountered in their respective clinical practice scenarios of PWSMI with co-morbid chronic physical conditions such as cardiovascular diseases and diabetes. Although research has argued that mental health medications and modifiable risk factors are largely responsible for their physical chronic conditions [2, 86,87], psychosocial factors such as stigma have been traditionally overlooked. Our findings document how healthcare professional’s stigmatizing attitudes and beliefs can directly impact their healthcare interventions. One example is how, among our participants, PWSMI were perceived as being different than patients with only physical conditions. Their narratives exemplified how this led some healthcare professionals in Puerto Rico to actively ignore their complaints or immediately refer them to a mental health professional despite their chief physical complaint. Literature has coined the term “diagnostic overshadowing” in order to account for this complex process of misattributing physical symptoms to an underlying mental illness [88]. Although research literature has emphasized the need for more research efforts to better understand this phenomenon, evidence to date suggests it appears to be a global occurrence [88,89]. However, the idea of *embuste* (big lie or fake) seems to play an important role in this process within the Puerto Rican context, with potential implications for other similar cultural context within Latin America and the Caribbean. As literature shows, Latinos/as emphasize non-biomedical interpretations of mental illnesses [90]. When interacting with PWSMI in their practices, the notion of mental illnesses as a non-biomedical and fake could be behind their dismissal of physical symptoms as these could also be seen as non-biomedical and un-real just like their mental illnesses. We understand that this is an aspect that needs to be further explored as it could offer a unique insight into some of the healthcare inequities Latinos/as with SMI encounter.

Other important findings also highlight the role of some cultural variables in the stigmatization process within the Puerto Rican context. For example, one participant explicitly mentioned that they abstain from directly asking if their patients in medical healthcare scenarios had any type of mental illness in order to avoid making them feel bad. Similarly, another participant mentioned regarding lack of disclosure from patients that they just hoped it hadn’t happened to them. This could be linked to the cultural value of *dignidad and respeto*. While this value emphasizes the intrinsic worth of all individuals, it can also lead to avoid discussing topics traditionally understood as private to a person, such as SMI [39, 45]. In addition, religion was mentioned in order to explain why PWSMI were perceived as outsiders. In this sense, religion was understood as a mechanism for keeping people within traditional cultural boundaries or norms. This is similar to the characteristic stigma function of “keeping people in” [23], which could be used to justify their attitudes and behaviors towards PWSMI. Finally, *familismo* seem to be linked to the perception of inappropriate care and support for their physical health needs. Participants expressed that families often do not support enough PWSMI. Interestingly, this is contrary to what literature has documented, particularly among Latinos/as living with a SMI for which families are the main role of social support [91, 92]. This finding could be evidence of exceeding expectations regarding the role of families in healthcare of PWSMI. Most importantly, these findings evidence the role of some cultural values in the stigmatization process and its implications for the physical healthcare of PWSMI, which we believe could be potentially transferable to other cultural settings beyond Puerto Rico, such as Latin America and the Caribbean as recent research has begun to suggest [39,42].

As we mentioned in our introduction, the stigmatization process in healthcare encounters is comprised of interrelated levels. Some participants described how structural aspects made it difficult for them to appropriately address PWSMI’ healthcare needs despite their best intentions. For example, participants informed us about the lack of appropriate training offered to work with PWSMI within medical settings in their respective medical and nursing programs. These findings reflect recent literature documenting the lack of training for primary care physicians and nurses in Puerto Rico regarding physical healthcare of PWSMI [93]. Furthermore, they also identified that the lack of training during their education also extends to continuing education after graduation. This led them to experience negative emotions when working with PWSMI in their medical practices, and in some instances forced them to neglect addressing vital aspects of routine healthcare interactions, such as the patient’s mental health history or their lithium levels. Thus, this lack of training seems to be driving the process of stigmatization in healthcare settings. Our results suggest healthcare professionals in our context lack basic knowledge about SMI, but also about how to address the physical needs of PWSMI. This is a troublesome issue for PWSMI’s medical healthcare. When healthcare professionals with little training and stigmatizing attitudes interact with PWSMI, stigmatizing behaviors can manifest leading to an atmosphere of mistrust, substandard healthcare and ultimately to this population’s elevated mortality rates as some participants also suggested. In addition, participants explained how institutional policies for PWSMI such as delayed insurance approval, negatively impacted the treatment and ultimately patient adherence. Furthermore, fragmented care fostered a lack of communication between physicians and mental healthcare providers, becoming a barrier for adequate treatment. When these structural aspects interact with the previously mentioned levels, PWSMI are systematically stigmatized in their healthcare interactions with healthcare professionals. These interactions can lead them to feel threatened and detached from their providers, which are vital aspects for effective interventions presumably worldwide, but particularly among Latinos/as with SMI [18].

### Limitations

There are some limitations to our study. Firstly, our participants practiced their profession in private institutions. Thus, our findings only reflect health professionals who practice in these scenarios and not those in public healthcare settings. This is important, as the patients they encounter in these contexts are usually those who have private insurance or economical means to cover their healthcare. Secondly, because participants preferred the interviews in their respective practicing scenarios, difficulties such as colleagues calling them to attend an emergency or an appointment were encountered. These might have resulted in a less than optimal participation and shortened quotations as interviewees had to divide their attention between their practice and the interviewed. This is certainly a methodological challenge that needs to be taken into consideration working with practicing healthcare professionals who have little time and a convoluted schedule. Thirdly, all our participants were interviewed only once. Thus, this limited the in-depth of the interviews and thus of findings. Lastly, because our participants were recruited via promotional flyers provided to them by key gatekeepers, some of them could have agreed to participate because of their interest in the study. Thus, some of the responses could have included some socially desirable information, which could impact our findings. However, in spite of these limitations, our study represents an important effort to understand and document the stigmatization process in healthcare interactions among an underrepresented population in the literature, practicing healthcare professionals [94].

## Conclusions

Our results evidence the existence of stigmatization towards PWSMI among practicing healthcare professionals in Puerto Rico. Furthermore, they point out the need to address the multilevel process of stigmatization and how its interrelationship specifically impacts physical healthcare interactions. They also evidence the need for urgent comprehensive research and intervention agenda to eliminate the health inequities encountered by PWSMI. Our study shows that healthcare professionals should be a priority in these endeavors. We understand that this work represents a starting point that could potentially inform subsequent research on stigma reduction globally, but more particularly across Latin America and the Caribbean. Although preliminary, our results suggest several issues that could potentially inform these future interventions efforts in this context. Specifically, these results suggest a need to focus on: (1) addressing cultural values and notions that might impact how PWSMI healthcare is being provided, (2) addressing practicing healthcare professional’s cognitions and emotion management related to PWSMI, (3) enhancing their clinical skills and competencies when working with PWSMI, (4) integrating SMI physical healthcare needs in the medical school and nursing school curriculum and training, and (5) revising institutional policies regarding medical care of PWSMI. These recommendations are a small but important effort for developing specific strategies to foster stigma free health encounters and eliminate health disparities among this population in the Puerto Rican context. The urgency to do so is a matter of life or death.

## Supporting information

S1 Fig Interview guide. (DOCX)

## References

[1] National Institutes of Mental Health. [Internet]. 2009. Available from: https://www.nimh.nih.gov/health/statistics/mental-illness.shtml

[2] De Hert M, Dekker J, Wood D, Kahl K, Holt R, Möller H. Cardiovascular disease and diabetes in people with severe mental illness position statement from the European Psychiatric Association (EPA), supported by the European Association for the Study of Diabetes (EASD) and the European Society of Cardiology (ESC). European Psychiatry. 2009;24(6):412–424. 10.1016/j.eurpsy.2009.01.005 19682863

[3] Lawrence D, Kisely S. Review: Inequalities in healthcare provision for people with severe mental illness. Journal of Psychopharmacology. 2010;24(4_suppl):61–68. 20923921 10.1177/1359786810382058 PMC2951586

[4] Walker E, McGee R, Druss B. Mortality in Mental Disorders and Global Disease Burden Implications. JAMA Psychiatry. 2015;72(4):334 10.1001/jamapsychiatry.2014.2502 25671328 PMC4461039

[5] Druss B, Zhao L, Von Esenwein S, Morrato E, Marcus S. Understanding Excess Mortality in Persons With Mental Illness. Medical Care. 2011;49(6):599–604. 10.1097/MLR.0b013e31820bf86e 21577183

[6] Thornicroft G. Physical health disparities and mental illness: the scandal of premature mortality. British Journal of Psychiatry. 2011;199(06):441–442. 22130744 10.1192/bjp.bp.111.092718

[7] Kisely S, Crowe E, Lawrence D. Cancer-Related Mortality in People with Mental Illness. 2013; 70 (2): 209–217. 10.1001/jamapsychiatry.2013.278 23247556

[8] Carliner H, Collins P, Cabassa L, McNallen A, Joestl S, Lewis-Fernández R. Prevalence of cardiovascular risk factors among racial and ethnic minorities with schizophrenia spectrum and bipolar disorders: a critical literature review. Comprehensive Psychiatry. 2014;55(2):233–247. 10.1016/j.comppsych.2013.09.009 24269193 PMC4164219

[9] Hellerstein D, Almeida G, Devlin M, Mendelsohn N, Helfand S, Dragatsi D et al Assessing Obesity and Other Related Health Problems of Mentally Ill Hispanic Patients in an Urban Outpatient Setting. Psychiatric Quarterly. 2007;78(3):171–181. 10.1007/s11126-007-9038-y 17417734

[10] Ortega A, Feldman J, Canino G, Steinman K, Alegría M. Co-occurrence of mental and physical illness in US Latinos. Social Psychiatry and Psychiatric Epidemiology. 2006;41(12):927–934. 10.1007/s00127-006-0121-8 17013767 PMC2791952

[11] Cordero D. Preocupa retrato de salud mental en Puerto Rico [Internet]. Metro. 2017 [cited 10 August 2018]. Available from: https://www.metro.pr/pr/noticias/2017/05/03/preocupa-retrato-salud-mental-puerto-rico.html

[12] Salud Mental en Puerto Rico. In: Departamento de Salud. http://www.salud.gov.pr/Documents/AcreditacióndelDepartamentodeSalud/PRDoHCHSA(DraftArt)SaludMental.pdf

[13] Alegría M, Canino G, Shrout P, Woo M, Duan N, Vila D et al Prevalence of Mental Illness in Immigrant and Non-Immigrant U.S. Latino Groups. American Journal of Psychiatry. 2008;165(3):359–369. 10.1176/appi.ajp.2007.07040704 18245178 PMC2712949

[14] Alegría M, Mulvaney-Day N, Torres M, Polo A, Cao Z, Canino G. Prevalence of Psychiatric Disorders Across Latino Subgroups in the United States. American Journal of Public Health. 2007;97(1):68–75. 10.2105/AJPH.2006.087205 17138910 PMC1716243

[15] Daviglus ML, Talavera GA, Avilés-Santa ML, Allison M, Cai J, Criqui MH, et al Prevalence of Major Cardiovascular Risk Factors and Cardiovascular Diseases Among Hispanic/Latino Individuals of Diverse Backgrounds in the United States. JAMA. 2013;308(17):1775–84. 10.1001/jama.2012.14517 PMC3777250 23117778

[16] Daviglus ML, Pirzada A, Durazo-Arvizu R, Chen J, Allison M, Avilés-Santa L, et al Prevalence of Low Cardiovascular Risk Profile Among Diverse Hispanic/Latino Adults in the United States by Age, Sex, and Level of Acculturation: The Hispanic Community Health Study/Study of Latinos. Journal of the American Heart Association. 2016;5(8). 10.1161/JAHA.116.003929 PMC5015308 27543802

[17] Go AS, Mozaffarian D, Roger VL, Benjamin EJ, Berry JD, Borden WB, et al Heart Disease and Stroke Statistics—2013 Update: A Report From the American Heart Association. Circulation. 2013;127(1). 10.1161/CIR.0b013e31828124ad PMC5408511 23239837

[18] Cabassa LJ, Gomes AP, Meyreles Q, Capitelli L, Younge R, Dragatsi D, et al Primary Health Care Experiences of Hispanics with Serious Mental Illness: A Mixed-Methods Study. Administration and Policy in Mental Health and Mental Health Services Research. 2013;41(6):724–36 10.1007/s10488-013-0524-2 PMC4000574 24162079

[19] Hatzenbuehler ML, Phelan JC, Link BG. Stigma as a Fundamental Cause of Population Health Inequalities. American Journal of Public Health. 2013;103(5):813–21. 10.2105/AJPH.2012.301069 23488505 PMC3682466

[20] Link B, Hatzenbuehler ML. Stigma as an Unrecognized Determinant of Population Health: Research and Policy Implications. Journal of Health Politics, Policy and Law. 2016;41(4):653–73. 10.1215/03616878-3620869 27127258

[21] Dijker AJM, Koomen W. Stigmatization, tolerance and repair: an integrative psychological analysis of responses to deviance Cambridge: Cambridge University Press; 2009.

[22] Link BG, Phelan JC. Conceptualizing Stigma. Annual Review of Sociology. 2001; 27: 363–385.

[23] Phelan JC, Link BG, Dovidio JF. Stigma and prejudice: One animal or two? Social Science & Medicine. 2008;67(3):358–67. 18524444 10.1016/j.socscimed.2008.03.022 PMC4007574

[24] Cook JE, Purdie-Vaughns V, Meyer IH, Busch JT. Intervening within and across levels: A multilevel approach to stigma and public health. Social Science & Medicine. 2014;103:101–9. 24513229 10.1016/j.socscimed.2013.09.023

[25] Stangl A, Earnshaw V, Logie C, Brakel W, Simbayi L, Barré I, Dovidio J. The Health Stigma Discrimination Framework: A Global, Crosscutting Framework to Inform Research, Intervention Development and Policy on Health Related Stigmas. BMC Medicine. 2019; 17; 31 10.1186/s12916-019-1271-3 30764826 PMC6376797

[26] Brakel W, Cataldo J, Grover S, Kohrt B, Nyblade L, Stockton M, Wouters E, Yang L. Out of the Silos: Identifying Crosscutting Features of Health-Related Stigma to Advance Measurement and Intervention. BMC Medicine. 2019: 17; 13 10.1186/s12916-018-1245-x 30764817 PMC6376667

[27] Koschorke M, Evans-Lacko S, Sartorius N, Thornicroft G. Stigma in Different Cultures In Gaebel W, Rössler W, Sartorius N. (Eds). The Stigma of Mental Illness–End of the Story? Springer; 2017; 65–82.

[28] Link BG, Yang LH, Phelan JC, Collins PY. Measuring Mental Illness Stigma. Schizophrenia Bulletin. 2004;30(3):511–41. 10.1093/oxfordjournals.schbul.a007098 15631243

[29] Yang LH, Kleinman A, Link BG, Phelan JC, Lee S, Good B. Culture and stigma: Adding moral experience to stigma theory. Social Science & Medicine. 2007;64(7):1524–35. 17188411 10.1016/j.socscimed.2006.11.013

[30] Link BG, Phelan JC, Bresnahan M, Stueve A, Pescosolido BA. Public conceptions of mental illness: labels, causes, dangerousness, and social distance. American Journal of Public Health. 1999;89(9):1328–33. 10.2105/ajph.89.9.1328 10474548 PMC1508784

[31] Smith V, Reddy J, Foster K, Asbury ET, Brooks J. Public perceptions, knowledge and stigma towards people with schizophrenia. Journal of Public Mental Health. 2011;10(1):45–56.

[32] Wood L, Birtel M, Alsawy S, Pyle M, Morrison A. Public perceptions of stigma towards people with schizophrenia, depression, and anxiety. Psychiatry Research. 2014;220(1–2):604–8. 10.1016/j.psychres.2014.07.012 25064387

[33] Mashiach-Eizenberg M, Hasson-Ohayon I, Yanos PT, Lysaker PH, Roe D. Internalized stigma and quality of life among persons with severe mental illness: The mediating roles of self-esteem and hope. Psychiatry Research. 2013;208(1):15–20. 10.1016/j.psychres.2013.03.013 23570969 PMC3665740

[34] Vogel DL, Bitman RL, Hammer JH, Wade NG. Is stigma internalized? The longitudinal impact of public stigma on self-stigma. Journal of Counseling Psychology. 2013;60(2):311–6. 10.1037/a0031889 23421775

[35] West ML, Yanos PT, Smith SM, Roe D, Lysaker PH. Prevalence of Internalized Stigma among Persons with Severe Mental Illness. Stigma Research and Action. 2011;1(1). 10.5463/sra.v1i1.9 PMC3144559 21804951

[36] Corrigan PW, Larson JE, Rüsch N. Self-stigma and the “why try” effect: impact on life goals and evidence-based practices. World Psychiatry. 2009;8(2):75–81. 10.1002/j.2051-5545.2009.tb00218.x 19516923 PMC2694098

[37] Whitley R. Social Defeat or Social Resistance? Reaction to Fear of Crime and Violence Among People with Severe Mental Illness Living in Urban ‘Recovery Communities.’ Culture, Medicine, and Psychiatry. 2011;35(4):519–35. 10.1007/s11013-011-9226-y 21701942

[38] Peluso ÉDTP Blay SL. Community perception of mental disorders. Social Psychiatry and Psychiatric Epidemiology. 2004;39(12):955–61. 10.1007/s00127-004-0820-y 15583902

[39] Mascayano F, Tapia T, Schilling S, Alvarado R, Tapia E, Lips W, et al Stigma toward mental illness in Latin America and the Caribbean: a systematic review. Revista Brasileira de Psiquiatria. 2016;38(1):73–85. 10.1590/1516-4446-2015-1652 27111703 PMC7115468

[40] Corrigan PW, Torres A, Lara JL, Sheehan L, Larson JE. The Healthcare Needs of Latinos with Serious Mental Illness and the Potential of Peer Navigators. Administration and Policy in Mental Health and Mental Health Services Research. 2016;44(4):547–57. 10.1007/s10488-016-0737-2 PMC5997453 27236458

[41] Rivera-Segarra E, Rivera G, López-Soto R, Crespo-Ramos G, Marqués-Reyes D. Stigmatization Experiences among People Living with Borderline Personality Disorder in Puerto Rico. The Qualitative Report. 2014;19(30):1–18.

[42] Caqueo-Urízar A, Boyer L, Urzúa A, Williams D. Self-Stigma in Patients with Schizophrenia: A Multicentric Study from Three Latin-American Countries. Social Psychiatry and Psychiatric Epidemiology. 2019; 10.1007/s00127-019-01671-4 30806726

[43] Sabogal F, Marín G, Otero-Sabogal R, Marín BV, Perez-Stable EJ. Hispanic Familism and Acculturation: What Changes and What Doesnt? Hispanic Journal of Behavioral Sciences. 1987;9(4):397–412.

[44] Villatoro AP, Morales ES, Mays VM. Family culture in mental health help-seeking and utilization in a nationally representative sample of Latinos in the United States: The NLAAS. American Journal of Orthopsychiatry. 2014;84(4):353–63. 10.1037/h0099844 24999521 PMC4194077

[45] Andrés-Hyman RC, Ortiz J, Añez LM, Paris M, Davidson L. Culture and clinical practice: Recommendations for working with Puerto Ricans and other Latinas(os) in the United States. Professional Psychology: Research and Practice. 2006;37(6):694–701.

[46] Torres JB, Solberg VSH, Carlstrom AH. The myth of sameness among Latino men and their machismo. American Journal of Orthopsychiatry. 2002;72(2):163–81. 10.1037/0002-9432.72.2.163 15792057

[47] Lawrence RE, Brent D, Mann JJ, Burke AK, Grunebaum MF, Galfalvy HC, et al Religion as a Risk Factor for Suicide Attempt and Suicide Ideation Among Depressed Patients. The Journal of Nervous and Mental Disease. 2016;204(11):845–50. 10.1097/NMD.0000000000000484 26894320 PMC4990512

[48] Yang LH, Chen F-P, Sia KJ, Lam J, Lam K, Ngo H, et al “What matters most:” A cultural mechanism moderating structural vulnerability and moral experience of mental illness stigma. Social Science & Medicine. 2014;103:84–93. 24507914 10.1016/j.socscimed.2013.09.009

[49] Pescosolido BA, Boyer CA. The American Health Care System Beginning the Twenty-First Century with High Risk, Major Challenges, and Great Opportunities In: Cockerham W, editor. The New Blackwell Companion to Medical Sociology. Oxford, U.K.: Wiley-Blackwell; 2010 p. 391–411.

[50] Schulze B. Stigma and mental health professionals: A review of the evidence on an intricate relationship. International Review of Psychiatry. 2007;19(2):137–55. 10.1080/09540260701278929 17464792

[51] Clement S, Schauman O, Graham T, Maggioni F, Evans-Lacko S, Bezborodovs N, Morgan C, Rüsch N, Brown J, Thornicroft G. What is the Impact of Mental Health-Related Stigma on Help-Seeking? A Systematic Review of Quantitative and Qualitative Studies. Psychological Medicine. 2015; 45; 11–27. 10.1017/S0033291714000129 24569086

[52] Henderson C, Noblett J, Parke H, Clement S, Caffrey A, Gale-Grant O, Schulze B, Druss B, Thornicroft G. Mental Health-Related Stigma in Health Care and Mental Health-Care Settings. Lancet Psychiatry. 2014; 1; 467–482 10.1016/S2215-0366(14)00023-6 26361202

[53] Kopera M, Suszek H, Bonar E, Myszka M, Gmaj B, Ilgen M, et al Evaluating Explicit and Implicit Stigma of Mental Illness in Mental Health Professionals and Medical Students. Community Mental Health Journal. 2014;51(5):628–34. 10.1007/s10597-014-9796-6 25535045 PMC4475542

[54] Stone E, Nawei L, Daumit G, Linden S, McGinty E. General Medical Clinicians’ Attitudes Towards People with Serious Mental Illness: A Scoping Review. Journal of Behavioral Health Services & Research. 2019: 1–23. 30887413 10.1007/s11414-019-09652-w PMC7251232

[55] Rao H, Mahadevappa H, Pillay P, Sessay M, Abraham A, Luty J. A study of stigmatized attitudes towards people with mental health problems among health professionals. Journal of Psychiatric and Mental Health Nursing. 2009;16(3):279–84. 10.1111/j.1365-2850.2008.01369.x 19291157

[56] Nordt C, Rössler W, Lauber C. Attitudes of Mental Health Professionals Toward People With Schizophrenia and Major Depression. Schizophrenia Bulletin. 2006; 32 (4); 709–714. 10.1093/schbul/sbj065 16510695 PMC2632277

[57] Pascal Hengartner M, Adrade Loch A, Lorea Lawson F, Bevilacquia F, Wang YP, Rössler W, Farid Gattaz W. Attitudes of Mental Health Professionals Towards Persons With Schizophrenia: A Transcultural Comparison Between Switzerland and Brazil. Rev Psiq Clin. 2012; 39 (4); 115–121.

[58] Andrade Loch A, Pascal Hengartner M, Bevilacqua Guarniero F, Lorea Lawson F, Wang YP, Farid Gattaz W, Rössler W. The more information, the more negative stigma towards schizophrenia: Brazilian general population and psychiatrists compared. Psychiatry Research. 2013; 205 (3); 185–191. 10.1016/j.psychres.2012.11.023 23266022

[59] Oliveira S, Esteves F, Carvalho H. Clinical Profiles of Stigma Experiences, Self-Esteem and Social Relationships Among People with Schizophrenia, Depressive, and Bipolar Disorders. Psychiatry Research. 2015; 229; 167–173. 10.1016/j.psychres.2015.07.047 26208983

[60] Ellison N, Mason O, Scior K. Bipolar Disorder and Stigma: A Systematic Review of the Literature. Journal of Affective Disorders. 2013; 151; 805–820. 10.1016/j.jad.2013.08.014 24135506

[61] Knaak S, Mantler E, Szeto A. Mental Illness-Related Stigma in Healthcare: Barriers to Access and Care and Evidence-Based Solutions. Healthcare Management Forum. 2017; 30 (2); 111–116. 10.1177/0840470416679413 28929889 PMC5347358

[62] Rivera-SegarraE, Rosario-Hernández E, Carminelli-Corretjer P, Tollinchi-Natali N, Polanco-Frontera N. Suicide Stigma Among Medical Students in Puerto Rico. Int J Environ Res Public Health. 2018; 15; 1366. 10.3390/ijerph15071366 PMC6068937 29966228

[63] Murphy A, O’Reilly C, Ataya R, Doucette S, Martin-Misener R, Rosen A, Gardner D. A Survey of Canadian and Australian Pharmacists’ Stigma of Suicide. SAGE Open Medicine. 2019; 7; 1–9. 10.1177/2050312118820344 PMC6350138 30728964

[64] Nutt R, Gilchrist G, Marsa Sambola F, Baldacchino A. Staff Regard Toward Working with Patients with Co-morbid Depression and Substance Misuse: An Exploratory Study. Heroin Addict Relat Clin Probl. 2017.

[65] Gilchrist G, Moskalewicz J, Slezakova S, Okruhlica L, Torrens M, Vajd R, Baldacchino A. Staff Regards Towards Working with Substance Users: A European Multi-Centre Study. Addiction. 2011; 106 (6): 1114–1125. 10.1111/j.1360-0443.2011.03407.x 21320230

[66] van Boekel LC, Browers EP, van Weeghel J, Garretsen HF. Healthcare Professionals’ Regards Towards Working with Patients with Substance Use Disorders: Comparision of Primary Care, General Psychiatry and Specialist Addiction Services. Drug and Alcohol Dependence. 2014 134; 92–98. 10.1016/j.drugalcdep.2013.09.012 24099970

[67] Varas-Díaz N, Santiago Negrón S, Neilands T, Cintrón Bou F, Malavé Rivera S. Stigmatization of Ilicit Drug Use Among Puerto Rican Health Professionals in Training. PR Health Sciences Journal. 2010; 29 (2); 109–116. PMC2877284 20496525

[68] Nyblade L, Stockton M, Giger K, Bond V, Eckstrand M, McLean R, Mitchell E, Nelson L, Sapag J, Siraprapasiri T, Turan J, Wouters E. Stigma in Health Facilities: Why it Matters and how can we Change it. BMC Medicine. 2019; 17; 25 10.1186/s12916-019-1256-2 30764806 PMC6376713

[69] Sickel A, Seacat J, Nabors N. Mental Health Stigma: Impact on Mental Health Treatment Attitudes and Physical Health. Journal of Health Psychology. 2016; 24 (5); 586–599. 10.1177/1359105316681430 27909162

[70] Polanco-Frontera N, Cajigas-Vargas I, Rivera-Segarra E, Varas-Díaz N, Santos-Figueroa A, Rosario-Hernández E. Estigma hacia problemas de salud mental entre profesionales de la salud en adiestramiento en Puerto Rico. Salud & Sociedad. 2013;4(3):250–63.

[71] Williams G, Elliot E. Exploring Social Inequalities in Health: The Importance of Thinking Qualitatively In: Bourgeault I, Dingwall R, de Vires R, editors. The Sage Handbook of Qualitative Methods in Health Research. Thousand Oaks: SAGE Publications Ltd; 2013 p. 106–22.

[72] Denzin N, Lincoln YS. The Discipline And Practice Of Qualitative Research In: Denzin N, Lincoln YS, editors, The Sage Handbook of Qualitative Research. Thousand Oaks: SAGE Publications Ltd; 2011 p. 1–20.

[73] Ross CA, Goldner EM. Stigma, negative attitudes and discrimination towards mental illness within the nursing profession: a review of the literature. Journal of Psychiatric and Mental Health Nursing. 2009;16(6):558–67. 10.1111/j.1365-2850.2009.01399.x 19594679

[74] Rapley T. The (extra)ordinary practices of qualitative interviewing In: Gubrium J, Holstein A, Marvasti A, McKinney K, editors. The SAGE Handbook of Interview Research: The Complexity of the Craft. 2nd ed. Thousand Oaks: SAGE Publications; 2012 p. 541–55.

[75] Kaisar K. Protecting Confidentiality In: Gubrium J, Holstein A, Marvasti A, McKinney K, editors. The SAGE Handbook of Interview Research: The Complexity of the Craft. 2nd ed. Thousand Oaks: SAGE Publications; 2012 p. 457–464.

[76] Cook K. Stigma and the Interview Encounter In: Gubrium J, Holstein A, Marvasti A, McKinney K, editors. The SAGE Handbook of Interview Research: The Complexity of the Craft. 2nd ed. Thousand Oaks: SAGE Publications; 2012 p. 333–344.

[77] Morse JM. Critical Analysis of Strategies for Determining Rigor in Qualitative Inquiry. Qualitative Health Research. 2015;25(9):1212–22. 10.1177/1049732315588501 26184336

[78] Guest G, Bunce A, Johnson L. How Many Interviews Are Enough? An Experiment with Data Saturation and Variability. Field Methods. 2006;18(1):59–82.

[79] Braun V, Clarke V. Using thematic analysis in psychology. Qualitative Research in Psychology. 2006;3(2):77–101.

[80] Fereday J, Muir-Cochrane E. Demostrating Rigor Using Thematic Analysis: A hybrid Approach of Inductive and Deductive Coding and Theme Development. International Journal of Qualitative Methods. 2006; 5 (1); 80–92.

[81] Decuir-Gunby JT, Marshall PL, Mcculloch AW. Developing and Using a Codebook for the Analysis of Interview Data: An Example from a Professional Development Research Project. Field Methods. 2010;23(2):136–55.

[82] Phelan JC, Lucas JW, Ridgeway CL, Taylor CJ. Stigma, status, and population health. Social Science & Medicine. 2014;103:15–23. 10.1016/j.socscimed.2013.10.004 PMC4091623 24507907

[83] Corrigan PW, Mittal D, Reaves CM, Haynes TF, Han X, Morris S, et al Mental health stigma and primary health care decisions. Psychiatry Research. 2014;218(1–2):35–8. 10.1016/j.psychres.2014.04.028 24774076 PMC4363991

[84] de Hert M, Correll CU, Bobes J, Cetkovich-Bakmas M, Cohen D, Asai I, et al Physical illness in patients with severe mental disorders: Prevalence, impact of medications and disparities in health care. World Psychiatry. 2011;10(1):52–77. 10.1002/j.2051-5545.2011.tb00014.x 21379357 PMC3048500

[85] McCloughen A, Foster K, Huws-Thomas M, Delgado C. Physical health and wellbeing of emerging and young adults with mental illness: An integrative review of international literature. International Journal of Mental Health Nursing. 2012;21(3):274–88. 10.1111/j.1447-0349.2011.00796.x 22533335

[86] Gray R. Physical health and mental illness: A silent scandal. International Journal of Mental Health Nursing. 2012;21(3):191–2. 10.1111/j.1447-0349.2012.00832.x 22533325

[87] Reis AF. Antipsychotic drugs and metabolic syndrome: can we prevent it? Revista Brasileira de Psiquiatria. 2007;29(1):9–10. 10.1590/s1516-44462007000100005 17435920

[88] Jones S, Howard L, Thornicroft G. ‘Diagnostic overshadowing’: worse physical health care for people with mental illness. Acta Psychiatrica Scandinavica. 2008;118(3):169–71. 10.1111/j.1600-0447.2008.01211.x 18699951

[89] Shefer G, Henderson C, Howard L, Murray J, Thornicroft G. Diagnostic Overshadowing and Other Challenges Involved in the Diagnostic Process of Patients with Mental Illness Who Present in Emergency Departments with Physical Symptoms–A Qualitative Study. PLOS One. 2014; 9 (11): e111682 10.1371/journal.pone.0111682 25369130 PMC4219761

[90] Carpenter-Song E, Chu E, Drake R, Ritsema M, Smith B, Alverson H. Ethno-Cultural Variations in the Experience and Meaning of Mental Illness and Treatment: Implications for Access and Utilization. Transcultural Psychiatry. 2010;47(2):224–51. 10.1177/1363461510368906 20603387

[91] Magana SM, Ramirez-Garcia J, Hernandez MG, Cortez R. Psychological Distress Among Latino Family Caregivers of Adults With Schizophrenia: The Roles of Burden and Stigma. Psychiatric Services. 2007;58(3):378–84. 10.1176/appi.ps.58.3.378 17325112 PMC2396526

[92] Rivera-Segarra E, Fernández A, Camacho-Acevedo Z, Ortiz N, López-Robledo Y. ‘Ahí Batallando’: the psychosocial impact of psychosis among Puerto Rican family caregivers. Journal of Family Studies. 2015;22(3):240–55.

[93] Instituto de Estadísticas de Puerto Rico. Informe sobre los sistemas de Salud Mental en Puerto Rico. 2016. http://www.who.int/mental_health/evidence/puerto_rico_whoaims_report.pdf

[94] Farrelly S, Jeffery D, Rüsch N, Williams P, Thornicroft G, Clement S. The link between mental health-related discrimination and suicidality: service user perspectives. Psychological Medicine. 2015;45(10):2013–22. 10.1017/S0033291714003158 25678059

