# Supplementary material for: “That’s all Fake”: Health professionals stigma and physical healthcare of people living with Serious Mental Illness
Source: PLoS One. 2019 Dec 18;14(12):e0226401. doi: 10.1371/journal.pone.0226401 (PMC6919598; doi:10.1371/journal.pone.0226401)
Supplement: S1 Fig — (DOCX) [file pone.0226401.s001.docx]

**S1 Fig. Interview Guide.docx**.

***This is a translated version of the Spanish semi-structured interview guide.**

I want to thank you for your interest in participating from this interview. During our time together I will ask you some questions regarding your knowledge about serious mental illness, your perceptions about those living with a serious mental illness and your work experiences with this population. Do you have any additional question? Let me know when can we begin our interview.

1. What do SMI’s means to you? What specific of conditions can you think of?
2. What does [bring examples from previous response: i.e. Schizophrenia, Bipolar Disorder] mean to you? Clarify if needed.
3. Have you ever had any clinical experience providing services to people diagnosed with SMI (mention specific examples brought up by participant).
4. How were these experiences? (Ask for specific examples).
5. Do you know people in your personal life who have SMI?
6. What do you think are they main mental health related needs of this population?
7. What do you think are the main medical needs of this population?
8. What do you think are the main social needs of this population?
9. How do you usually address these needs?
10. How did your training prepare you to address this population’s needs?
11. How has your continuing education prepared you to address this population’s needs?
12. In addition, we would like to receive input from you regarding how to best manage a case in which a person with a mental illness comes to your office for a routine checkup.
    1. Juan is a 28 year old man who comes to your clinic. After collecting the history, the mother informs that Juan’s problems began after losing his job as a butcher 3 years ago. Juan lives with his mother who is currently unemployed and received government’s help to sustain themselves. After losing his job, Juan began to present mood changes, usually being uneasy, suspicious, and when we didn’t felt well, it was hard to understand him. His condition worsened about six months ago when we has verbally abusive to his family and friends. Juan often said that they were mocking him and that he had instructions from God to eliminate those who mocked him. During that time, he said that he. Was being watched through the TV and phones. Three months ago, Juan had an episode in which he destroyed furniture, TV, cellphone and car lights in order to intercept a message from those who were watching him. In light of this, his mother called the police and he was translated to a hospital via an involuntary admission. We has hospitalized for three weeks. His mother reports that as of today, although he is not taking medications, he is better. It is because of this that she was able to convince him to go for a routine checkup, even though he didn’t wanted to go.
    2. Jennifer is a 33 year old woman that works as a nurse and comes for a routine visit. You notice that she reports feeling sad and hopeless and that sometimes she doesn’t want to get out of bed. This has been increasingly difficult after her partner broke up with her. Jennifer refers she is managing by going to church.
